# Family practitioners' top medical priorities when managing patients with multimorbidity: a cross-sectional study

**DOI:** 10.3399/bjgpopen18X101622

**Published:** 2019-01-23

**Authors:** Lilli Herzig, Yolanda Mueller, Dagmar M Haller, Andreas Zeller, Stefan Neuner-Jehle, Anouk Déruaz-Luyet, Christine Cohidon, Sven Streit, Bernard Burnand, Jean-Christophe Zuchuat

**Affiliations:** 1 Chief of Research Department, Department of Family Medicine, General Medicine and Public Health Centre, University of Lausanne, Lausanne, Switzerland; 2 Chief of Research Department, Department of Family Medicine, General Medicine and Public Health Care Centre, University of Lausanne, Lausanne, Switzerland; 3 Chief of Research Department, Primary Care Unit, Faculty of Medicine, University of Geneva, Geneva, Switzerland; 4 Chief of Institution, Centre of Primary Health Care, University of Basel, Basel, Switzerland; 5 Research Collaborator, Institute of Primary Care, University of Zürich, Zürich, Switzerland; 6 Projects Chief, Department of Family Medicine, General Medicine and Public Health Centre, University of Lausanne, Lausanne, Switzerland; 7 Research Collaborator, Institute of Primary Care, Department of Family Medicine, General Medicine and Public Health Centre, University of Lausanne, Lausanne, Switzerland; 8 Research Collaborator, Institute of Primary Health Care (BIHAM), University of Bern, Bern, Switzerland; 9 Head of Department, Institute of Social and Preventive Medicine, Lausanne University Hospital, Lausanne, Switzerland; 10 Statistician, Department of Family Medicine, General Medicine and Public Health Centre, University of Lausanne, Lausanne, Switzerland

**Keywords:** primary care, chronic disease, multimorbidity, epidemiology, prioritisation

## Abstract

**Background:**

Managing multiple chronic and acute conditions in patients with multimorbidity requires setting medical priorities. How family practitioners (FPs) rank medical priorities between highly, moderately, or rarely prevalent chronic conditions (CCs) has never been described. The authors hypothesised that there was no relationship between the prevalence of CCs and their medical priority ranking in individual patients with multimorbidity.

**Aim:**

To describe FPs’ medical priority ranking of conditions relative to their prevalence in patients with multimorbidity.

**Design & setting:**

This cross-sectional study of 100 FPs in Switzerland included patients with ≥3 CCs on a predefined list of 75 items from the International Classification of Primary Care 2 (ICPC-2); other conditions could be added. FPs ranked all conditions by their medical priority.

**Method:**

Priority ranking and distribution were calculated for each condition separately and for the top three priorities together.

**Results:**

The sample contained 888 patients aged 28–98 years (mean 73), of which 48.2% were male. Included patients had 3–19 conditions (median 7; interquantile range [IQR] 6–9). FPs used 74/75 CCs from the predefined list, of which 27 were highly prevalent (>5%). In total, 336 different conditions were recorded. Highly prevalent CCs were only the top medical priority in 66%, and the first three priorities in 33%, of cases. No correlation was found between prevalence and the ranking of medical priorities.

**Conclusion:**

FPs faced a great diversity of different conditions in their patients with multimorbidity, with nearly every condition being found at nearly every rank of medical priority, depending on the patient. Medical priority ranking was independent of the prevalence of CCs.

## How this fits in

The management of multimorbidity in primary care is complex, and FPs need to set medical priorities. FPs’ rankings of medical priorities in the management of multimorbidity had never been described previously.

No relationship was found between the medical priority ranking of different chronic or acute conditions and their prevalence. 

A better understanding of how FPs set their medical priorities could lead to better agreement with patients’ priorities, which can be different, and to the creation of guidelines adapted to their daily practice.

## Introduction

Multimorbidity, defined as the co-occurrence of more than two or three chronic conditions (CCs) in one person, is increasingly prevalent as global populations age.^1–3^ Multimorbidity is often present in well-described patterns of highly prevalent CCs.^4–9^ The most frequent patterns associate cardiovascular diseases and their risk factors, metabolic syndromes (such as diabetes and obesity), pulmonary diseases, psychological disorders (such as depression and anxiety), and osteoarthritic pain.^10^ However, the prevalence of multimorbidity varies greatly depending on definitions, the CCs included, study design, populations, measures, and outcomes.^1,11–16^

Co-managing a patient’s multiple chronic and non-chronic conditions with different levels of severity is often extremely challenging for FPs. They must therefore try to set medical priorities, as the co-management of every condition present during the same consultation is often impossible.^17^ FPs are trained to focus first on the clinical problems which pose the highest risk of mortality or morbidity to the patient (such as heart diseases and diabetes), and this may be their way of setting priorities.^18^

The innumerable combinations of different chronic and acute conditions found in primary care make the management of patients with multimorbidity non-standard and often complex.^19^ Most clinical practice guidelines provide recommendations for dealing with some of the most prevalent CCs,^20^ but FPs face an extreme diversity of combinations of CCs; some of these appear individually quite infrequently, but they have a high medical priority and therefore require an in-depth assessment of how to treat them in the context of other CCs. Due to their relative uncommonness, such rare CCs are usually not integrated into studies on multimorbidity but are a daily problem for FPs. Newer guidelines integrate the most frequent CCs, but this still does not solve every problem for FPs, who cannot simply apply clinical recommendations generically to a specific individual’s situation.^20–23^ It was therefore hypothesised that the highly prevalent and well-described CCs were not always FPs’ top medical priorities in primary care.

The present study, therefore, aimed to describe FPs' medical priority ranking of CCs in relation to their prevalence for each individual multimorbid patient in a primary care study sample.

## Method

The present study is a secondary analysis of the cross-sectional Multi-Morbidity in Family Medicine (MMFM) study of patients with multimorbidity in primary care in Switzerland.^24^ The detailed study protocol and first results have been published elsewhere.^9,25^ Briefly, the MMFM study involved a convenience sample of 100 FPs across five large regions of Switzerland. Eligible participants were patients with multimorbidity aged >18 years, suffering from ≥3 CCs on a predefined list of 75 items identified from the International Classification of Primary Care 2 (ICPC-2).^25,26^ FPs could subsequently add other conditions affecting patients. When available, these additional conditions were recoded with their usual ICPC-2 classifications by the research team. Each patient included gave their written informed consent to participate in the study.

FPs were asked to list the conditions by order of medical importance for each patient on the day of their inclusion in the study (that is, how patients presented to their FPs on that day). In the present analysis, positions in this ordering are called the medical priority ranking. 

Data are available at the Department of Family Medicine, General Medicine and Public Health Centre, University of Lausanne. 

## Statistical analyses

### Definitions

In the following analyses, three categories of CCs were defined based on their prevalence in the sample and their presence or not on the predefined list of 75 items:

1. on the list with a prevalence ≥5% was termed 'highly prevalent';
2. on the list with a prevalence <5% was termed 'moderately prevalent'; and
3. not on the list was termed 'other' and included all the other conditions recorded by the FPs. This category contained CCs, but also acute conditions, combinations of conditions (such as asthma and chronic obstructive pulmonary disease listed together), or social problems. The prevalence of items was not calculated in this heterogeneous category, but the medical priority rankings which FPs gave to each condition were analysed.

### Analyses

The analyses involved three steps.

#### Step one

The medical priority ranking of each CC on the list was extracted (categories 1 and 2). Each CC’s prevalence within the sample was calculated, as well as the median and IQR of the medical priority ranking. The prevalence and median priority ranking of CCs were illustrated using a scatterplot diagram. Prevalence was represented using a logarithmic scale to enhance readability. Spearman’s rho and Kendal’s tau were calculated to assess the correlation between a CC’s prevalence and the median level of its medical priority ranking; the null hypothesis of an absence of association was also tested.

Furthermore, for each patient, the relative priority ranking was calculated by taking the ratio between the medical priority rank for a given CC and the maximal number of mentioned CCs for this patient. The following formula was used: 1-(Rank-1)/ (Total number of mentioned CCs-1)

The results were expressed in per cent, where 100% was the highest possible priority and 0% the lowest.

#### Step two

The distribution of medical priority rankings for every condition in each of the three categories of CCs was illustrated using bar charts.

#### Step three

The research team analysed which conditions FPs retained as the top three medical priorities for every individual patient. Each patient was classified into one of three groups: the first group’s top three ranked medical priorities were all 'highly prevalent' CCs (only category 1). The second group’s top three ranked medical priorities included ≥1 'moderately prevalent' CC, but there were no 'other' condition (categories 1 and 2). The third group’s top three ranked medical priorities included ≥1 'other' condition (categories 1, 2, 3).

Furthermore, to illustrate and better understand the distribution of FPs’ medical priority rankings, some specific subcategories of the ICPC-2 were analysed: cardiovascular and psychiatric conditions, because of their high prevalence and appearance in numerous clinical recommendations; and malignant neoplasms, because they are not usually integrated into clinical recommendations, but they usually have a high medical priority.

All statistics were computed using the R statistical language (version 3.3.2). Figures were made using the ggplot2 package (version 2.2.1).

## Results

### General

The MMFM study’s general results have been published elsewhere.^9^ Briefly, the sample contained 888 patients, with a mean age of 73 years (range 28–98), and 48.2% were male. They were prescribed 8 medications on average (mean 7.7; SD 3.5). The median number of CCs was 5 (range 1–16; IQR 6–9) for CC categories 1 and 2 combined. By adding category 3 ('others'), the median number of all conditions rose to 7 (range 3–19; IQR 6–9).

### Type and prevalence of the conditions mentioned

Overall, FPs used 74/75 CCs on the predefined list (only 'malignant neoplasm of the nervous system' was not used), of which 27 had a prevalence ≥5%. All the FPs added at least one 'other' condition at least once, and 90% of them added ≥6 different 'other' conditions, with an average of 21 'other' conditions mentioned per FP across all their included patients. Only 15% of the patients had exclusively CCs from the predefined list. The 100 FPs recorded a total of 336 different conditions: 74 from the predefined list, and 262 'others'.

The median medical priority ranking for each CC on the list of 75 is described in Table 1 in relation to its prevalence (categories 1 and 2). One was the highest medical priority ranking and 19 the lowest.

**Table 1:** Medical priority ranking (absolute and relative) for each chronic condition on the list and its prevalence (categories 1 and 2). 1 was the highest medical priority ranking and 19 the lowest for the absolute ranking, whereas 100% was the highest medical priority for the relative ranking. Chronic condition labels adapted from ICPC-2.

Chronic conditions		Cases in the sample, *n*	Prevalence, %	Absolute medical priority ranking					Relative medical priority ranking, %				
				Median	Quantiles				Median	Quantiles			
					High	0.25	0.75	Low		High	0.25	0.75	Low
Highly prevalent chronic conditions	Hypertension uncomplicated	460	51.8	3	1	2	5	15	60	100	80	33	0
	Risk factor cardiovascular	380	42.8	6	1	4	8	14	32	100	56	11	0
	Obesity	283	31.9	5	1	3	7	13	44	100	67	22	0
	Depressive	238	26.8	4	1	2	6	15	52	100	75	25	0
	Osteoarthritis, knee	223	25.1	5	1	3	6	15	50	100	71	25	0
	Atrial fibrillation	210	23.6	2	1	2	3	16	80	100	100	61	0
	Pain, general	205	23.0	5	1	2	8	15	36	100	86	0	0
	Diabetes, non insulin-dependent	204	22.9	2	1	1	4	12	76	100	100	50	0
	Atherosclerosis	167	18.8	5	1	3	8	15	50	100	71	17	0
	Ischaemic heart without angina	164	18.5	2	1	1	4	13	83	100	100	61	0
	Osteoporosis	163	18.4	5	1	3	7	15	43	100	71	20	0
	Hypertension, complicated	159	17.9	4	1	2	6	15	57	100	80	33	0
	Osteoarthritis, hip	153	17.3	5	1	3	7	13	50	100	75	26	0
	Chronic obstructive pulmonary disease	126	14.2	3	1	1	6	12	71	100	100	37	0
	Neuropathy	112	12.6	5	1	3	7	15	43	100	71	17	0
	Hearing complaint	110	12.4	6	1	4	8	12	25	100	42	13	0
	Cerebrovascular disease	106	11.9	4	1	2	7	13	50	100	88	20	0
	Ischaemic heart with angina	104	11.7	2	1	1	3	11	88	100	100	74	0
	Gout	100	11.2	5	1	4	7	13	33	100	50	17	0
	Diabetes, insulin-dependent	91	10.2	2	1	1	3	10	88	100	100	71	0
	Elevated blood pressure	90	10.1	4	1	3	7	11	40	100	67	20	0
	Asthma	86	9.6	4	1	3	6	14	53	100	72	32	0
	Irritable bowel disease	84	9.4	4	1	3	7	15	39	100	69	20	0
	Tobacco abuse	73	8.2	5	1	3	7	14	29	100	69	9	0
	Rheumatoid arthritis	68	7.6	2	1	1	6	14	82	100	100	31	0
	Incontinence	62	7.0	6	1	4	7	12	40	100	63	17	0
	Chronic alcohol abuse	49	5.6	4	1	2	6	11	58	100	88	25	0
	Prostate neoplasm	49	5.6	4	1	2	6	11	50	100	83	13	0
Slightly prevalent chronic conditions	Macular degeneration	42	4.8	5	1	4	6	15	43	100	66	21	0
	Involuntary movement	41	4.7	5	1	4	7	14	37	100	57	16	0
	Chronic bronchitis	40	4.5	5	1	3	6	12	43	100	75	22	0
	Migraine	38	4.3	5	1	3	7	14	25	100	67	17	0
	Chronic skin ulcer	27	3.1	5	1	3	7	12	50	100	67	21	0
	Memory disturbance	26	3.0	5	1	2	8	16	60	100	80	20	0
	Trauma	26	3.0	4	1	3	7	10	44	100	75	29	0
	Breast female, neoplasm	25	2.8	4	1	2	5	15	57	100	88	38	0
	Colorectal neoplasm	25	2.8	2	1	1	5	9	70	100	100	45	0
	Retinopathy	25	2.8	6	1	5	8	13	32	100	41	27	0
	Bladder neoplasm	22	2.5	3	1	2	6	12	50	100	79	34	0
	Parkinsonism	22	2.5	2	1	1	4	10	80	100	100	50	0
	Personality disorder	22	2.5	2	1	2	5	12	78	100	89	30	0
	Chronic enteritis	21	2.4	7	1	4	9	15	22	100	41	0	0
	Other blood neoplasm	21	2.4	4	1	1	6	11	63	100	100	40	0
	Epilepsy	19	2.2	5	2	4	6	13	43	86	50	20	0
	Phobia, compulsive	19	2.2	4	1	3	7	15	29	100	85	11	0
	Pulmonary heart disease	19	2.2	5	1	3	8	14	63	100	76	26	0
	Somatisation	18	2.0	6	1	3	7	14	52	100	70	14	0
	Dementia	17	1.9	4	1	2	6	8	57	100	89	35	0
	Bowel incontinence	16	1.8	5	1	3	9	11	33	100	71	23	0
	Medication abuse	16	1.8	5	1	3	6	10	64	100	80	16	0
	Multiple sclerosis	13	1.5	1	1	1	1	7	100	100	100	100	29
	Affective psychosis	11	1.2	4	1	2	8	10	69	100	93	27	0
	Blindness	11	1.2	6	1	3	8	12	36	100	80	19	0
	Bronchus lung neoplasm	11	1.2	1	1	1	6	6	100	100	100	62	29
	Deafness	10	1.1	6	3	4	7	10	27	75	58	0	0
	Other malignant neoplasm	10	1.1	5	1	3	8	13	15	100	56	10	0
	Drug abuse	8	0.9	5	1	3	8	11	42	100	82	7	0
	Lymphoma	8	0.9	6	1	3	9	11	33	100	77	16	0
	Anorexia bulimia	7	0.8	5	4	4	7	13	40	73	47	13	0
	Post-traumatic stress	6	0.7	4	4	4	6	10	51	80	63	23	0
	Kidney neoplasm	5	0.6	3	1	1	4	5	78	100	85	72	64
	Pain, face	5	0.6	8	2	2	9	9	36	89	80	0	0
	Psychosis, other	5	0.6	4	1	2	9	12	25	100	90	11	8
	Schizophrenia	5	0.6	1	1	1	3	6	80	100	100	59	55
	Thyroid neoplasm	5	0.6	5	1	2	6	8	53	100	83	25	13
	Poliomyelitis	4	0.5	6	2	3	9	10	57	86	75	31	0
	Cervix neoplasm	3	0.3	3	2	3	6	8	41	82	61	20	0
	Organic psychosis, other	3	0.3	7	4	6	9	11	25	45	35	13	0
	Other digestive neoplasm	3	0.3	4	4	4	7	9	42	73	57	27	11
	AIDS	2	0.2	5	3	4	5	6	44	60	52	36	29
	Pancreas neoplasm	2	0.2	2	1	2	3	3	92	100	96	88	85
	Stomach neoplasm	2	0.2	2	1	2	3	3	75	100	88	63	50
	Trigeminal neuralgia	2	0.2	6	3	5	8	9	46	71	59	33	20
	Mental retardation	1	0.1	1	1	1	1	1	100	100	100	100	100

**Figure 1:**
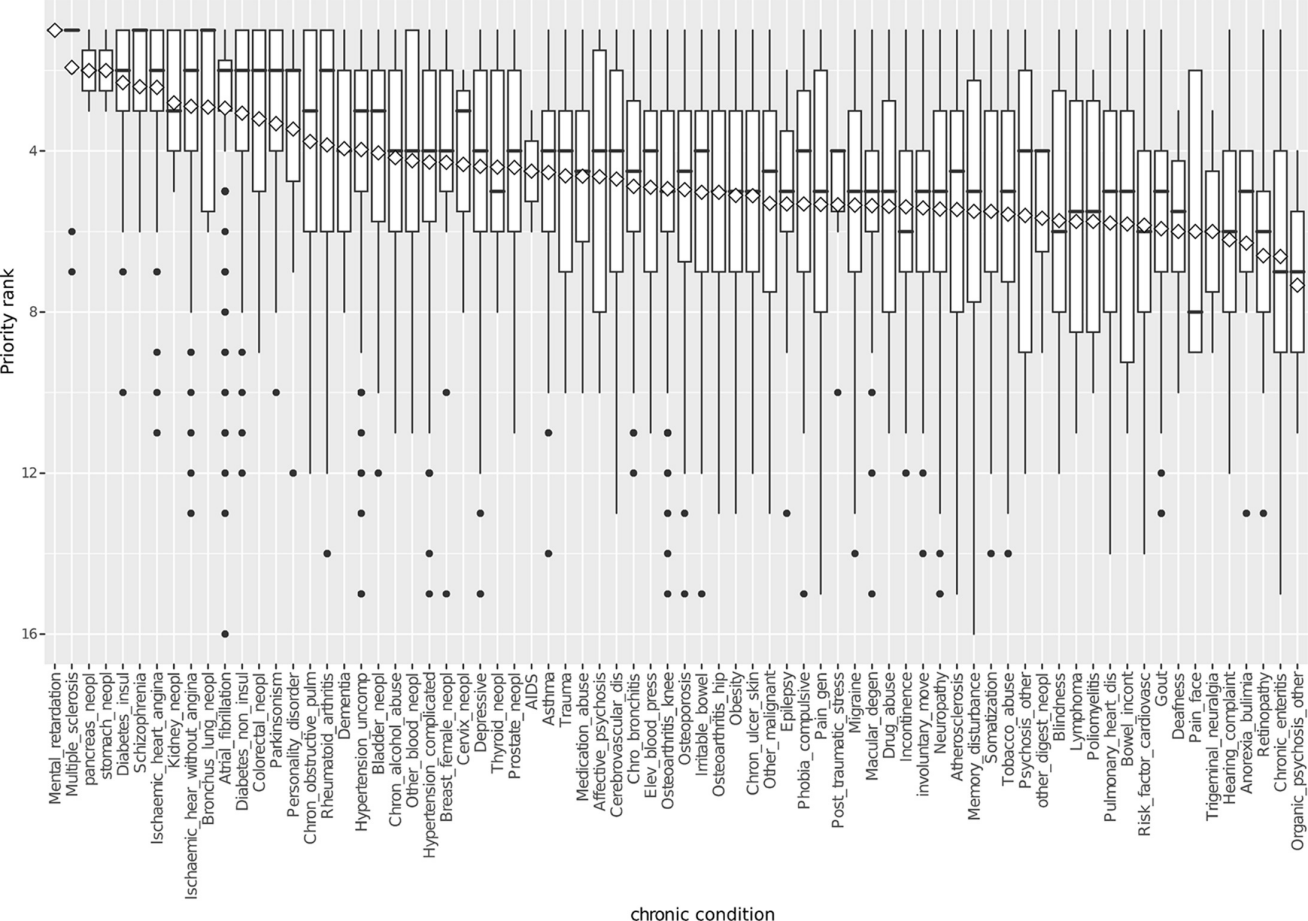
Boxplot of medical priority rank given by family practitioners to 74 chronic conditions (categories 1 and 2), sorted by mean rank (diamond)

For each of the 74/75 CCs (category 1), the medical priority rankings are most of the time highly variable (Figure 1). Even if a CC had a low median priority ranking, it could be ranked as a high priority for some patients, including a first, second, or third priority position. It was found that 64/74 CCs (86%) were ranked as the highest medical priority for ≥1 patient. However, no correlation was found between the prevalence of a given CC and its median ranking (Spearman's rho ≈ Kendal's tau ≈ -0.02; *P *value ≈ 0.8).

This variability is conserved, even if the relative ranks are used instead of the absolute ranks (Table 1). Indeed, relative ranks also show that nearly all CCs could be found at nearly every place of the medical ranking made by the FP, whether the patient had three or 19 CCs.

**Figure 2:**
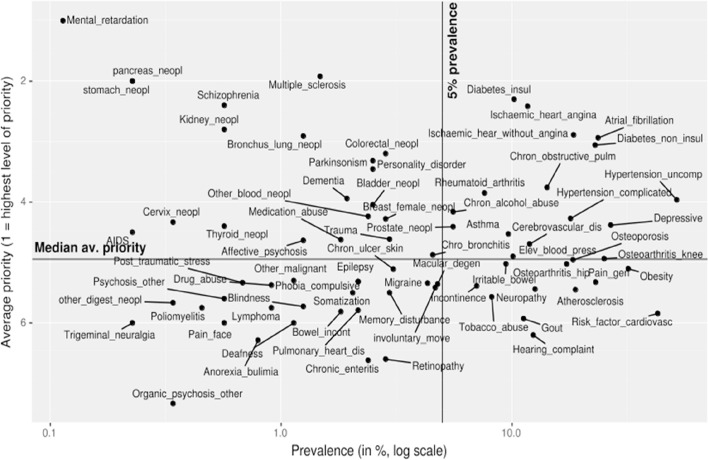
Scatterplot of correlation between prevalence and medical priority ranking

To illustrate the absence of correlation between prevalence and medical priority ranking, a scatterplot diagram was created for the CCs of categories 1 and 2 (Figure 2). Most rare CCs with high medical priority rankings (upper left quadrant) involved different ICPC-2 categories, without systematic distribution: *n* = 10 neoplasms from different systems, *n* = 6 psychological, and *n* = 2 neurological CCs. On the other side, high prevalence CCs with high medical priority rankings (upper right quadrant) most often concerned the ICPC-2 categories cardiovascular (*n* = 7 CCs), respiratory (*n* = 2 CCs), metabolic (*n* = 2 CCs), or psychological (*n* = 2 CCs).

### Types of conditions among the top three medical priorities

**Figure 3:**
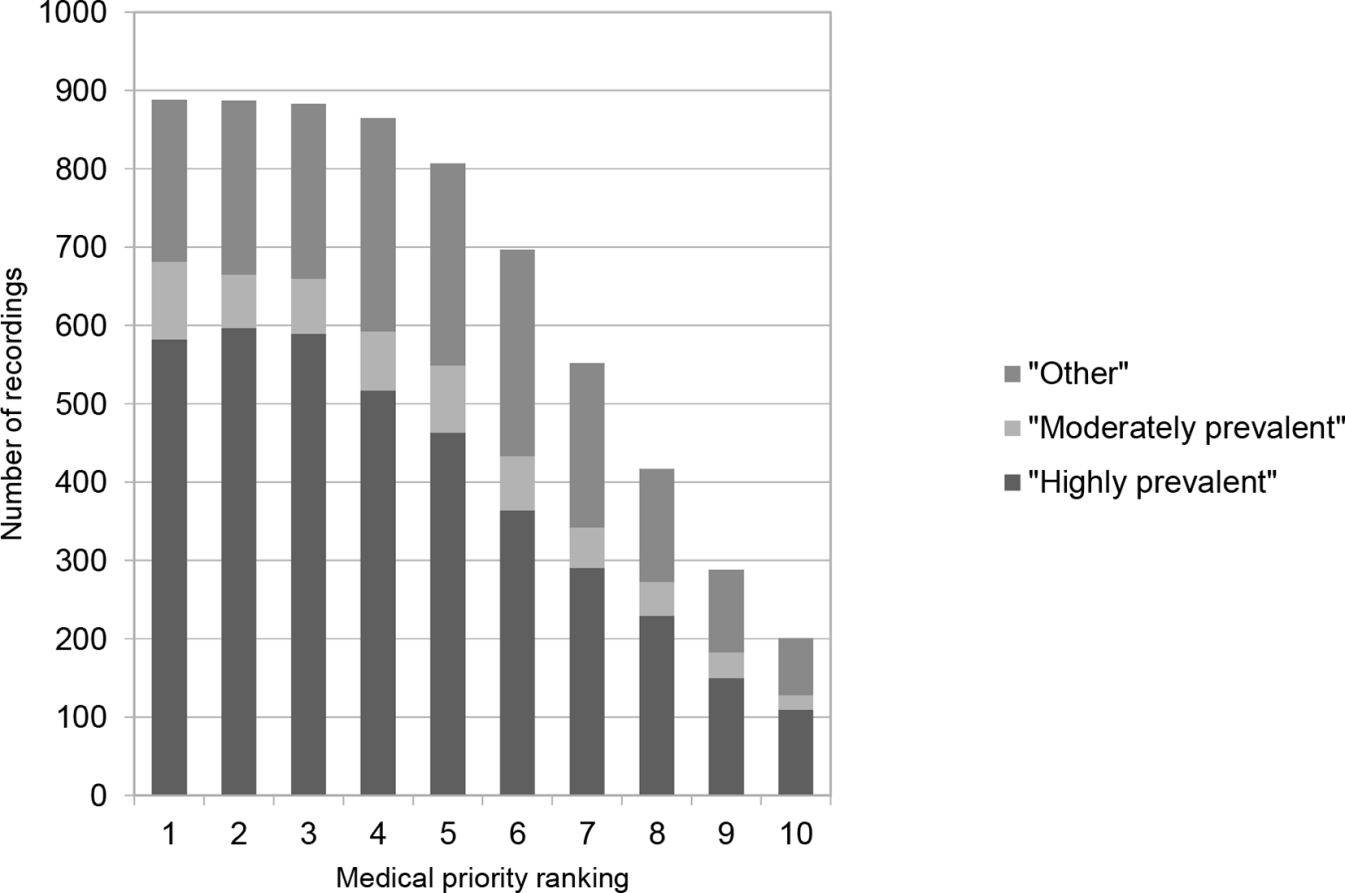
Relationship between medical priority ranking and category of conditions

Figure 3 illustrates the distribution of CC prevalence per medical priority ranking defined by FPs: a 'highly prevalent' CC (*n* = 27/75) was ranked as the first medical priority in 66% of cases, a 'moderately prevalent' CC was ranked first in 11% of cases. In the remaining 23%, FPs ranked an 'other' condition as the first medical priority. Response patterns for the second- and third-ranked medical priorities were similar.

**Figure 4:**
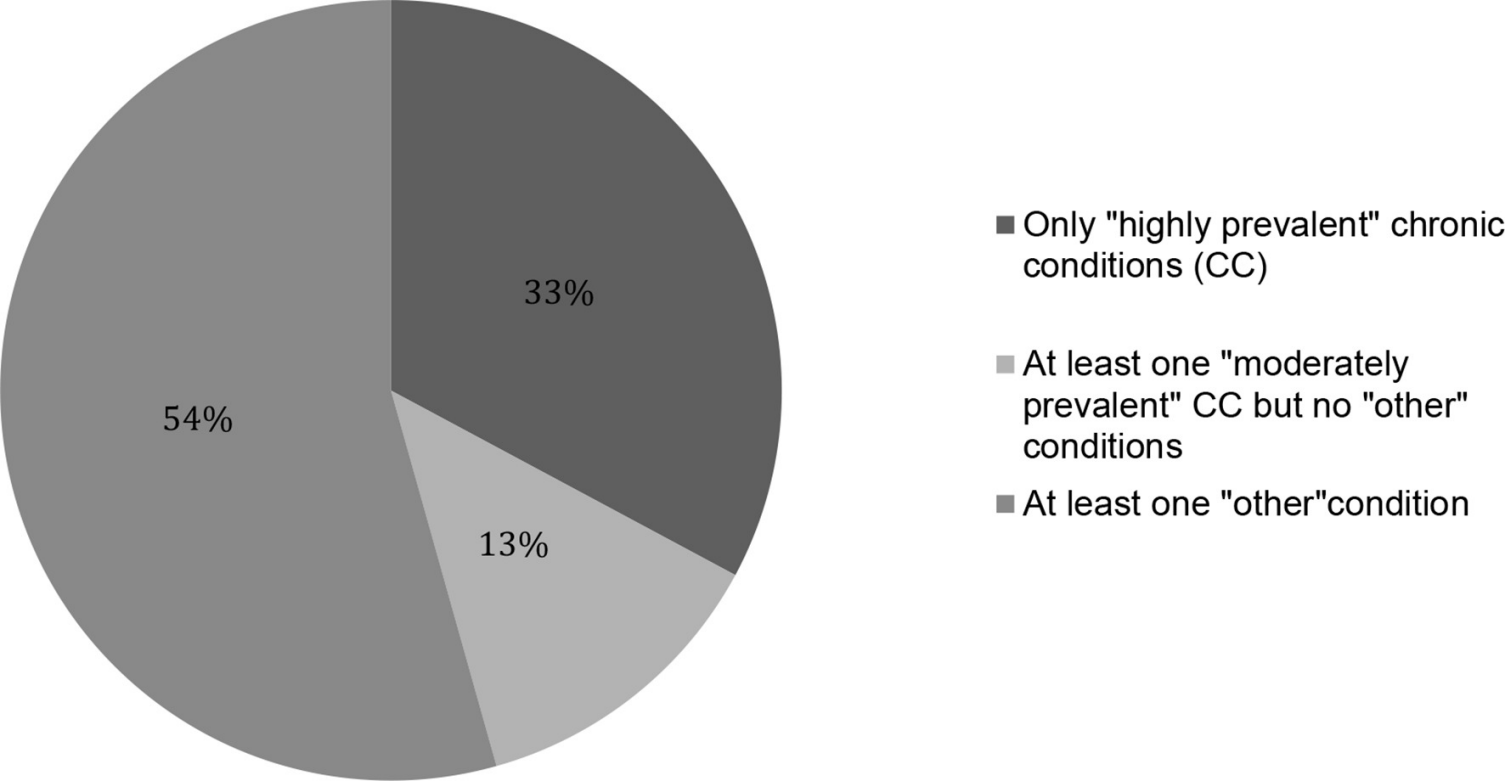
Distribution of the three categories of conditions among the top three ranking medical priorities

In 33% of patients, FPs ranked three of the 27 'highly prevalent' CCs (category 1) as their top three medical priorities. In 13% of patients, ≥1 'moderately prevalent' CC (category 2) was ranked among the top three medical priorities, but with no 'other' condition. In 54% of patients, FPs ranked an 'other' condition (category 3) among the top three medical priorities. This distribution is shown in Figure 4.

To better illustrate the absence of relationship between the prevalence of CCs and their medical priority ranking the following categories of the ICPC-2 were analysed.

A category K (cardiovascular condition) was recorded ≥1 time (median 2; IQR 1–3; max 6) in 89% of the patients (*n* = 792/888) and in 77% (*n* = 568/792) of them, FPs ranked ≥1 K condition among their top three medical priorities.

Thirty-nine per cent of patients had ≥1 category P (psychiatric): condition (*n* = 347/888), of whom 47% (*n* = 161/347) had ≥1 P condition cited among their top three medical priorities.

Of the overall sample, 17% had ≥1 malignant neoplasm ranked in their list of medical priorities (*n* = 156/888), of whom 27% (*n* = 43/156) had a neoplasm among their top three medical priorities.

## Discussion

### Summary

The present study analysed FPs’ medical priority rankings for every individual patient with multimorbidity in primary care. Two important results were found.

First, nearly all the conditions included were to be found at nearly every medical priority ranking given by the FPs. Indeed, no systematic distribution of medical priority rankings were found for any given set of CCs; rankings could be different for each patient. In other words, even if a condition generally had a low medical priority ranking, it could be ranked as a high medical priority for some patients, including in the first, second, or third positions.

Second, no correlation was found between medical priority ranking and the prevalence of a given condition: some rare CCs could have a very high medical priority ranking. For example, mental retardation was described only once, but it was that patient’s top medical priority. The rare CCs found in the present sample might not necessarily be found in other samples, but the authors nevertheless believe that disparate groups of rarely prevalent CCs with a high medical priority are surely to be found in all samples of patients with multimorbidity in primary care.

### Strengths and limitations

The present study’s main strength is that, to the best of the authors' knowledge, it was the first exploratory research on FPs’ rankings of the medical priorities attached to the different conditions of patients with multimorbidity in a primary care context.

Other strengths were the inclusion of patients with multimorbidity across large regions of Switzerland and of younger patients; most studies of multimorbidity investigate patients aged >65 or >80 years. Furthermore, a pre-defined list of 75 CCs was used as a framework, with the possibility of adding other conditions that FPs felt were relevant. Thus, the authors were able to describe the great diversity of conditions seen in primary care and provide a fuller picture of FPs’ daily practice.

The study has some limitations. First, a selection bias cannot be excluded as the inclusion criteria only concerned the 75 CCs on the predefined list. It cannot be certain that every condition not on this list was recorded. However, if there had been a selection bias, the authors would have expected the number of conditions seen and the importance of their distribution at the top of the medical priority rankings to be even higher, thus reinforcing the findings.

A second potential limitation is that medical priorities may change over time, depending on the presence of new acute or chronic conditions. The authors' interpretation of the duration of medical priorities was limited because of an absence of information about the severity of the conditions included, their evolution or the patients’ functional statuses. But as this is the first study describing FPs’ ranking of medical priorities, it would be interesting if further studies examined how these priorities change over time.

The difficulty inherent in comparing patients with multimorbidity with 3–19 different conditions is a third limitation. The dispersion of medical priority rankings may be different among patients with fewer CCs. Nevertheless, sub-analyses by patient subgroup, FPs’ demographic factors, or linguistic area did not change these analyses (data not shown). And sub-analyses of the relative medical priority ranking confirmed the authors' interpretation. Furthermore, it is believed that the highest ranking of medical priorities recorded by FPs were probably representative of patient’s reality that day, but that the rankings attributed to the lower-end priorities may have been more arbitrary (such as deciding whether a CC should be ranked 10 or 11).

### Comparison with the literature

An extensive literature search found no published studies to support the present results. However, some references, especially those evaluating complexity analyses, suggested the importance of exploring FPs’ opinions on medical priorities among the different CCs that patients present with.^27^

Thus, to the best of the authors' knowledge, the present study is the first to analyse FPs’ opinions on ranking medical priorities when managing patients with multimorbidity.

### Implications for practice

Developing better knowledge of what FPs deem to be medical priorities has two important implications for practice.

First, as some authors have mentioned, knowing FPs’ views on medical priorities would allow better comparisons with patients’ priorities, as they may be different.^28^ Indeed, patients’ priorities may more often be based on symptomatic problems, whereas FPs may prioritise clinical problems with a high risk of mortality or morbidity (such as heart diseases or diabetes).^18^ To illustrate this hypothesis, supported by the literature, this study showed, for example, that the vast majority of patients declared pain to be their most important complaint, yet painful conditions only appear in the central area of the scatterplot diagram, suggesting that pain is not the top medical priority for FPs.^29^ To develop better concordance and shared decision-making between patients’ and FPs’ priorities, it would therefore also be helpful to better understand how FPs prioritise CCs in patients with multimorbidity. Second, although the highly prevalent CCs found in patients with multimorbidity have been included in the clinical guidelines, this may still not be sufficient for primary care, where integrating treatments for highly, moderately, and rarely prevalent acute and chronic conditions is the norm, as this study confirmed.^15,20,22^ Furthermore, even the newest guidelines do not specify the medical priority of the different comorbidities included in them.

The inclusion of neoplasms (17% of cases in the present sample) in this study's analyses may also illustrate the importance of exploring medical priority rankings in relation to guidelines. A neoplasm may be a higher medical priority than hypertension, obesity, or silent risk factors and, indeed, neoplasms were ranked among the top three medical priorities in 27% of the patients presenting with them. However, no information was available about the severity or current state of any specific CC, therefore a cancer could be a low medical priority if it were in remission. Yet, even though a neoplasm can be among a patient’s top medical priorities, no guidelines were found in the literature targeting cancer that indicated how to manage it in patients with multimorbidity in primary care.

In conclusion, this was the first study to explore FPs’ rankings of different conditions by medical priority when managing patients with multimorbidity in primary care. It found no correlation between the prevalence of medical conditions and FPs’ rankings of their medical priority. In fact, there were a great variety of different conditions, and nearly every condition was found among at least one patient’s top three medical priorities. Furthermore, although highly prevalent CCs appear frequently in primary care, by definition, the ranking of moderately or rarely prevalent chronic or acute conditions among the top three medical priorities proved to be the norm, rather than the exception in the present study. Future studies should investigate how FPs determine the medical priorities of different conditions and whether they match with patients’ priorities. This could lead to better adapted guidelines for the management of patients with multimorbidity in primary care and to a better understanding of the gap between patients’ and FPs’ priorities.
